# Agricultural factor markets in Sub-Saharan Africa: An updated view with formal tests for market failure

**DOI:** 10.1016/j.foodpol.2016.09.015

**Published:** 2017-02

**Authors:** Brian Dillon, Christopher B. Barrett

**Affiliations:** a University of Washington, Evans School of Public Policy and Governance, United States; b Cornell University, Dyson School of Applied Economics and Management, United States

## Abstract

This paper uses the recently collected Living Standard Measurement Study–Integrated Surveys on Agriculture Initiative data sets from five countries in Sub-Saharan Africa to provide a comprehensive overview of factor market participation by agrarian households and to formally test for failures in rural markets. Under complete and competitive markets, households can solve their consumption and production problems separately, so that household factor endowments do not predict input demand. This paper implements a simple, theoretically grounded test of this separation hypothesis, which can be interpreted as a reduced form test of market failure. In all five study countries, the analysis finds strong evidence of factor market failure. Moreover, those failures appear general and structural, not specific to subpopulations defined by gender, geography, human capital, or land quality. However, we show that rural markets are not generally missing in an absolute sense, suggesting that market existence is less of a problem than market function.

## 1 Introduction

In the structural adjustment era of the 1980–90s, widespread belief in the efficiency of markets underpinned a broad transition away from government management and toward market liberalization in much of Sub-Saharan Africa (SSA). In the ensuing decade and a half, as it has become clear that liberalization *per se* was not sufficient to raise growth rates and rapidly reduce poverty throughout the region, attention has returned to market failures. Among the markets most widely believed to be failing or incomplete are the agricultural factor markets of SSA. And indeed, there are good reasons to suspect that rural markets are not functioning well in this region, as agricultural productivity and rates of modern input use lag far behind the rest of the world (World Bank, 2008). With the aim of stimulating productivity growth and reducing poverty, substantial resources are committed each year to programs aimed at improving the function of agricultural input and output markets in African economies.

To make appropriate policy choices in an atmosphere of potentially dysfunctional or imperfect markets it is important to distinguish between three cases. The first is a situation in which a market is truly *missing*, in the sense that exchange is legally prohibited, rendered infeasible by some non-market force, or impossible to undertake without the creation of a new regulatory or market-making institution. The second is a case in which a market is in operation but *failing* in the sense that exchange takes place at non-competitive prices, i.e., prices that do not equate marginal benefit and marginal cost. And the third situation is one in which a market is present and functioning at competitive prices, but welfare outcomes for some households are so low that the development community uses the mantle of “market failure” to motivate interventions aimed at improving wellbeing.

To illustrate, consider the following situation for a generic agricultural input. Suppose that the market for the input is hampered by high transaction costs, weak enforcement of contracts, and significant output risk – features common to rural economies in SSA. These forces could induce market failure by causing mismatches in supply and demand or underpinning the formation of oligopolies by a small number of active suppliers. But these features also increase suppliers’ costs, which shifts supply curves inward, raises equilibrium prices, and reduces trading volumes. If it is the latter case that pertains in a particular market, then low input use is the equilibrium outcome of competitive markets, even though it may be sub-optimal from a social perspective.

This distinction is essential to policy design, because the instruments to fix missing markets are not the same as those to introduce competition to non-competitive markets or to increase the welfare of certain agents in a well-functioning market. If evidence suggests that factor markets are missing in their entirety, the appropriate step is likely to create markets by assigning property rights, removing restrictions on certain forms of exchange, or providing public goods that make exchange feasible (e.g., information, roads, agricultural research stations). If, by contrast, there is robust evidence of market exchange but evidence indicates that agricultural factor markets are not competitive, then there is a case for interventions directly targeted at the sources of market failure. These might take the form of policies to improve contract enforcement, to end collusion, or to lower costs of production through investment in public goods and services such as physical (e.g., power, roads, telecommunications, water) and institutional (e.g., grades and standards) infrastructure. Finally, if a market exists and operates at market-clearing prices, but outcomes are considered sub-optimal from a social perspective, then greater attention should be paid to increasing the value *above* current market prices of the land and labor that constitute the primary endowments of the poorest households. Policies in this domain may include training and education, subsidies, taxes and transfer to mitigate endowment inequalities, or temporary assistance to stimulate learning-by-doing or take advantage of agglomeration externalities.

The aim of this paper is to provide an over-arching, updated view of agricultural land and labor markets in sub-Saharan Africa, in light of the above categorization. The analysis is in two parts. First, we document the patterns of market participation by agricultural households using recently collected, nationally representative data from five countries: Ethiopia, Malawi, Niger, Tanzania, and Uganda. We show with descriptive evidence that, in fact, a large share of farmers transact in agricultural labor and/or land markets, as well as in the market for other, related goods.^1^ These markets plainly exist and are used extensively. While transaction costs may prohibit market participation by some households in some markets (de Janvry et al., 1991), it would clearly be incorrect to characterize the land and labor markets of SSA as *missing* in a broad sense.

Second, we use a well-established, reduced form approach to test for market failures (Benjamin, 1992, Udry, 1999). The test is grounded in the standard model of the agricultural household (Singh et al., 1986), which makes explicit the prediction that under complete and competitive markets households can make decisions about production and consumption separately. This is widely known as the *separation hypothesis*. If the separation hypothesis holds, households behave as if they allocate resources to maximize farm profits first, and then make consumption choices conditional on the budget set that results from farm profit maximization. Endowments and preferences affect consumption, but not first-stage production choices. This generates the testable prediction that the household’s labor endowment is not predictive of labor demand on the family farm when markets are functioning well.^2^

We test this prediction for the five study countries. Our findings strongly reject the null hypothesis of complete and competitive markets. Although there is some between-country variation in the elasticity of farm labor demand with respect to the number of working age household members, our main estimates lie in the range 0.32–0.53 for all countries. We further show that the pattern of market failures is general and structural, related neither to the gender or education of the household head nor to geographic characteristics such as the distance to roads or large population centers. We do find that in a few cases the degree of market failure varies between agro-ecological zones, suggesting that market performance across the region is related at least in part to agro-climatic factors outside households’ control (Binswanger-Mkhize and Savastano, 2014).

Although we implement the above test using the labor demand equations of household farms, the results of these tests do not reveal whether the underlying market frictions are in the market for labor or for some other good. Rejection of the separation hypothesis is evidence that *at least two* factor or output markets fail to clear at competitive prices (it is well known that resource flows and relative prices can adjust to accommodate one quasi-fixed or non-tradable factor of production – see Feder, 1985). However, if there are failures in at least two markets, then the separation hypothesis will generally be rejected even if the market in which the test is implemented is capable of functioning well (in the sense that prices and quantities can adjust to equate marginal benefit and marginal cost).^3^

Overall our findings suggest that while factor markets are not missing, even 15–20 years after structural adjustment there is reason to believe that widespread market failures in rural SSA lead to sub-optimal resource allocation. This impedes productivity growth and poverty reduction. Of course, not every market failure is sufficient to merit intervention, and determining which market imperfections should be targeted by governments and donors requires case-by-case analysis of the related costs and benefits (Holden and Binswanger, 1998). The contribution here is to use recent, nationally representative data and theoretically grounded tests to show that in recent years market *existence* is less of a problem than market *function*, and to characterize the extent of the latter at the national scale.

The work here contributes to two primary strands of literature. The first is the voluminous body of work on agricultural input markets in sub-Saharan Africa, to which we cannot do justice with a review. It does bear mentioning that our findings align with one of the central themes in Fafchamps (2004), which documents the widespread existence, richness, and adaptability of rural markets in SSA.

This paper also contributes to the recent body of work evaluating market function or testing the necessary conditions consistent with market failure in a variety of settings in sub-Saharan Africa. Not surprisingly, the findings in this literature are mixed. Berg (2013) uses anticipated changes in household income in South Africa to test for the presence of credit constraints. While he cannot reject that the observed patterns are due to precautionary savings, he does find strong indicative evidence of credit market failures. In the context of a multi-factorial randomized controlled trial, Karlan et al. (2014) find strong evidence for incomplete insurance markets among farmers in Ghana. Barrett et al. (2008) show with data from Côte d’Ivoire that significant differences exist between shadow wages derived from estimated production functions and local market wages paid the same workers, which can be interpreted as evidence of failure in multiple agricultural input markets. On the other hand, separate studies from Kenya and Malawi suggest that given the relative prices of outputs and fertilizer, subsidies may induce most farmers to apply fertilizer at levels well beyond that which is profitable, calling into question the degree to which input market failures are a binding constraint on productivity (Ricker-Gilbert et al., 2009, Sheahan, 2011). This paper augments these country-specific studies by applying a standardized, general test to recent, high quality data from five different countries.

The rest of the paper proceeds as follows. In the following section we briefly review the core theoretical model, the optimal conditional factor demand functions derived from that model that underpin the separation hypothesis, and the empirical specification of the reduced form test of that hypothesis. Data and descriptive statistics are presented in Section 3. Section 4 presents results, and Section 5 concludes.

## 2 Theory and empirical framework

In this section we outline a basic model of the agricultural household and emphasize the role played by the separation hypothesis. Our goal is only to describe the basic empirical framework, so we leave aside complicating factors that can be readily incorporated but that do not change the central implications of the model. The exposition in this section most directly follows Udry, 1999, Barrett et al., 2008, and is effectively a single period version of the model in LaFave and Thomas (2014).^4^

In a particular cropping year a household has a total labor endowment of $\overset{‾}{L}$, which it divides between leisure $L^{l}$, work on the household farm $L^{f}$, and supply of labor to the market, $L^{m}$. The household has preferences over consumption of goods, C, and leisure, $L^{l}$, represented by the strictly increasing, concave utility function $U(C\text{,}L^{l}|Z)$. The utility function is conditional on household characteristics *Z*, which includes endowments not explicitly denoted elsewhere. The household produces a single food commodity, *y*, for sale or consumption using a strictly increasing, concave production technology y=F(L,X,A)ε, where L represents total labor application, X is a vector of non-labor inputs, A represents land inputs, and the stochastic variable ε, which follows known distribution g(ε), represents exogenous agro-climatic factors such as pests and weather conditions. The household owns land $\overset{‾}{A}$ and rents in (net) land area $A^{r}$, the sum of which is total land in cultivation, A. The household can hire labor on the market, represented by $L^{d}$. Let $p_{x}$ be the vector of non-labor input prices, w be the market wage rate, $p_{A}$ be the price of land, and p be the price of the output, all of which are known to the household. Under these conditions the household’s expected utility maximization problem is:

(1) $${\text{Max}}_{C\text{,}L^{l}\text{,}L^{h}\text{,}L^{d}\text{,}L^{m}\text{,}A^{r}\text{,}X}{\text{E}}_{\varepsilon }[U(C\text{,}L^{l}|Z)]$$

subject to:

(2) $$\mathrm{pC}-{\mathrm{wL}}^{m}⩽\mathrm{pF}(L\text{,}X\text{,}A)\varepsilon -{\mathrm{wL}}^{d}-p_{x}X-p_{A}A^{r}$$

(3) $$L\equiv L^{f}+L^{d}$$

(4) $$A\equiv \overset{‾}{A}+A^{r}$$

(5) $$\overset{‾}{L}⩾L^{f}+L^{m}+L^{l}$$

(6) $$L^{l}\text{,}L^{f}\text{,}L^{d}\text{,}L^{m}\text{,}X\text{,}A\text{,}C⩾0$$

where the expectation is taken over g(ε).

Under standard assumptions about the utility function, weak inequalities (2), (5) bind at the solution. The problem can be solved by first choosing total agricultural labor demand L, non-labor inputs X, and land inputs A to maximize expected farm profit. This is represented by the right-hand side of the inequality in (2). The household then solves its expected utility maximization problem conditional on optimal profits. This is the essence of the separation hypothesis. With complete and competitive markets, the household can buy and sell labor, land and other inputs at exogenous, market-clearing prices, so that its production and consumption decisions can be examined as if they were distinct problems.

If the separation hypothesis holds, the solution to the household’s problem implies the following:

(7) $$\Pi^{*}(p\text{,}p_{x}\text{,}p_{A}\text{,}w)=\max_{A\text{,}X\text{,}L}{\text{E}}_{\varepsilon }[\mathrm{pF}(L^{*}\text{,}X^{*}\text{,}A^{*}|W)\varepsilon -{\mathrm{wL}}^{d*}-p_{x}X^{*}-p_{A}A^{*}]$$

(8) $$L^{*}=L(p\text{,}p_{x}\text{,}p_{A}\text{,}w|\varepsilon )$$

(9) $$A^{*}=A(p\text{,}p_{x}\text{,}p_{A}\text{,}w|\varepsilon )$$

(10) $$X^{*}=X(p\text{,}p_{x}\text{,}p_{A}\text{,}w|\varepsilon )$$

(11) $$C^{*}=C(p\text{,}p_{x}\text{,}p_{A}\text{,}w\text{,}\overset{‾}{L}\text{,}A^{o}|Z\text{,}\varepsilon )$$

where Eq. (7) is the profit function, Eqs. (8), (9), (10) are the input demand functions, and Eq. (11) is the consumption function.

Various tests based on Eqs. (7), (8), (9), (10) can be interpreted as tests of the underlying assumption of complete and competitive factor markets. As is clear in these equations, inputs depend only on exogenous factors if the separation hypothesis holds. This suggests a reduced form strategy for testing the separation hypothesis: include in the estimation of an input demand function any other variable that does not appear in Eqs. (7), (8), (9), (10) but that does appear in Eq. (11), i.e., any variable that affects consumption behavior but not production choices when markets are complete. Natural options are the household labor endowment or other household characteristics that should influence consumption patterns without impacting the household’s full income. A test of complete and competitive markets can be implemented as a test of the exclusionary restriction implied by the theory, that production input demands are invariant to household characteristics. In particular, labor demand on the farm should be invariant to household labor endowments, $\overset{‾}{L}$, measured as the number of working age adults. This is the intuition underlying most of the tests in Benjamin (1992). Following this approach, we focus on the restriction implied by Eq. (8), the conditional labor demand function of the household under the null hypothesis of complete and competitive markets.

To test the restriction implied by the separation hypotheses, we estimate regressions of total farm labor demand on prices, land and labor endowments, and household characteristics $Z_{h}$, using the following general specification:

(12) $$\log L_{h}=\alpha +\beta \log{\overset{‾}{L}}_{h}+\delta \log{\overset{‾}{A}}_{h}+\gamma Z_{h}+\phi \mathrm{Prices}+\mu_{h}$$

where (α,β,δ,γ,ϕ) represent coefficients, the subscript h indicates households, and μ is a mean zero, i.i.d., normally distributed error term. In this case the separation hypothesis is represented by the null hypothesis, $H_{0}:\beta =0$. Rejection of that null in favor of the alternate hypothesis, $H_{A}:\beta \neq 0$, implies rejection of the exclusion restriction that follows directly from the presence of complete and competitive markets.

We report OLS estimates of Eq. (12), separately for each of the five study countries. In focusing on this baseline specification we make a number of simplifying assumptions. In particular, we treat the endowments of land and household size as exogenous. Household size in our empirical tests is defined as the number of adults aged 15+. We do not further disaggregate household size by demographic characteristics of household members, opting instead to include demographic controls in $Z_{h}$. We ignore the role of supervisory household labor as a complement to hired labor. To adjust for possible productivity differences between children and adults, we scale the contribution of child workers on farm by 0.5. Finally, we ignore harvest period labor because it is generally interpreted as proportional to output, rather than an input to production.^5^

The test of the separation hypothesis implicit in Eq. (12) cannot be interpreted as a test of labor market failure specifically. It is well understood that multiple market failures are required to generate distortions in factor or output markets because relative prices – not absolute prices – are what matters in determining the efficient allocation of resources (Feder, 1985). For example, in this context the fact that production is risky and crop insurance is unavailable is not sufficient on its own to generate a rejection of the separation hypothesis. If, however, we reject the null hypothesis that the coefficient on household size is statistically indistinguishable from zero, we can conclude that some markets (potentially including markets for credit, insurance, or land) are failing. A detailed exploration of precisely *which* markets are failing is left to future analysis.

## 3 Data

The data for this paper are from the Living Standards Measurement Study and Integrated Surveys on Agriculture (LSMS-ISA) project, sponsored by the Bill and Melinda Gates Foundation and implemented by the national statistics offices of participating countries with technical expertise and oversight provided by the Development Research Group of the World Bank.^6^ These nationally representative data sets cover a comprehensive set of demographic, health, economic and agricultural topics. Although there is variation in survey content between countries, efforts were made to ensure as much cross-national comparability as possible in questionnaire design and coverage. Panel data are available for some countries and will be available for all study countries in the coming years, though in this paper we make use of only a single cross section for each country. For each country we restrict the sample to households that report cultivation of a positive amount of land during the season under study.

We use data from the five LSMS-ISA countries with sufficient information on agricultural labor demand: Ethiopia, Malawi, Niger, Tanzania, and Uganda. Because the hypotheses of interest to this paper relate to market function within a cultivation period, we have not combined data across multiple cropping seasons. Instead, we use data relevant for the major cropping season in a single, nearly contemporaneous wave of each of the data sets. These are the 2011 cropping season in Ethiopia; the 2008/2009 rainy season in Malawi; the 2010 rainy season in Niger; the 2010 long rainy season in Tanzania; and the first cropping season of 2010 in Uganda.

### 3.1 Household-level summary statistics

Table 1 shows summary statistics at the household level for all five study countries. In Ethiopia, the average household has 5.24 members of all ages, with slightly more males than females. Just over 80% of households are male-headed. Education levels among household heads are the second lowest of the study countries, at 1.61 years of education on average. Of the 2.81 acres owned by the average household, 76% is cultivated, 3% is left fallow, and 22% is used for all other purposes, including pastures, forest land, and renting out. The average household cultivates more than 11 plots, with each crop stand on a single parcel listed as a separate plot. Eighteen percent of cultivated acreage is described as rented or borrowed. Household allocate a majority of cultivated land to the production of staple grains – maize, sorghum, and teff – with significant allocation of remaining land to cash crops, pulses, and nuts.^7^

The next set of columns show similar statistics from the 2010–2011 Malawi survey. The Malawi survey includes over 10,000 households, only a quarter of which are designated as panel survey households. We restrict our analysis to the panel households because the labor demand modules given to cross-sectional and panel households are slightly different, and labor demand issues are central to the questions in this paper.

In Table 1 we see that households in Malawi are slightly (but not statistically significantly) smaller than those in Ethiopia, with 4.96 members on average. Household heads are far more educated on average (4.98 years of education) than in Ethiopia, and almost a quarter of households are female-headed. The average household owns 1.58 acres and owns or cultivates 2 separate plots.^8^ Of the acreage owned by households, only a very small fraction is listed as “rented out”. Finally, crop distributions in Malawi are heavily dominated by maize production, which accounts for 1.45 acres of cultivated land on average. Farmers primarily use the remaining land to cultivate pulses, nuts, and cash crops.^9^

In the middle of Table 1 we show summary statistics for the Niger sample. Niger is the poorest country in the study. Households are especially large, encompassing almost seven people, on average. Only 8% of household heads are female. Educational attainment by household heads is the lowest among study countries, averaging less than one year completed. Households report significantly greater land ownership than in the other study countries, with average holdings of almost 12 acres. Although this statistic is driven in part by a small number of large landowners, the median household owns 7.4 acres, which is still larger than the average acres owned in any other study country. Farming households in Niger cultivate almost all of the land that they own, and rent an average of 1.6 acres of additional land for cultivation. Cultivation is concentrated heavily in sorghum, millet, and beans, with relatively small amounts of land allocated to other crops.

The next set of columns in Table 1 provides summary statistics for the 2010–2011 data from Tanzania. As mentioned above, agricultural variables for Tanzania refer only to the long rainy season. Not surprisingly, the demographic characteristics of households in Tanzania are more similar to those for Malawi than Ethiopia or Niger. The average sample household from Tanzania has 5.55 members, with slightly more females than males. Household heads are 25% female, and have 4.58 years of education on average. Households in Tanzania report owning less land than those in Niger, but much more land than those in smaller Malawi or more populous Ethiopia: 5.31 acres on average, 81% of which is cultivated and 15% of which is fallow. Only 2% of owned land is described as “rented out”, while 14% of cultivated land is listed as rented in or borrowed. Maize is the primary crop in Tanzania, with 2.2 acres of maize grown by the average household. Land allocation to the other crop categories – other grains, rice, tubers (including cassava), pulses and nuts, cash crops, and other crops (including banana) – ranges from 0.31 to 0.57 acres on average.

Finally, in the rightmost columns of Table 1 we provide a similar set of summary statistics for Uganda. Households in Uganda have 6.64 members on average, again with slightly more females than males. Only 71% of households in Uganda are male-headed. Households own 3.29 acres on average, and own or cultivate 5.65 plots. Ninety percent of owned land is cultivated, 7% is fallow, and essentially none is described as “rented out”. Households report cultivating just under 3 acres on average, 79% of which is owned and 21% of which is described as rented in or borrowed. Tubers, including cassava, account for the largest proportion of cultivated acreage, followed by pulses/nuts and “other crops” (which includes all bananas). Only 0.41 acres of maize are cultivated on average.

### 3.2 Market participation: Summary statistics

In Table 2 we present sample statistics for participation in land markets by sample households. Across study countries there is surprising consistency in the pattern of renting or borrowing land for cultivation, with percentages ranging from 19.6% in Tanzania to 38.7% in Uganda. The percentage of sample households renting land in is much larger than that renting land out. There is likewise general consistency in the average amount of land rented or borrowed in, with estimates in all countries but Niger lying between 0.38 and 0.62 acres. In Niger the average household cultivated over two acres of rented or borrowed land. Average acres rented out is less than average acres rented in for all study countries, as one would expect in a sample restricted to households that cultivate, as the subsample of cultivators necessarily omits households that own but do not cultivate land, who are by definition net suppliers of rented land.

Turning to the market for agricultural laborers, Table 3 shows the percentage of households that report hiring workers for various non-harvest activities during the studied cultivation period. This is one area where there is substantial heterogeneity in the level of detail covered in the LSMS-ISA surveys, therefore cross-country comparisons should be made cautiously. The overall pattern of labor hiring is consistent across study countries, with approximately 30–50% of households hiring workers at some point during the cultivation season, not including the harvest. In Ethiopia and Tanzania, just under a third of households report hiring of some laborers (30.2% and 30.1%, respectively). For these two countries the distribution of hiring is generally consistent across activities, with the exception of very limited hiring for fertilizer application in Tanzania. In Malawi, Niger, and Uganda, over 40% of households hire workers. In Malawi and Niger the rate of hiring for non-harvest activities is almost twice that for the harvest. This is consistent with the perception that if labor constraints bind because of seasonality in demand, they are most likely to do so in the harvest period (Benjamin, 1992). The labor module in Uganda does not disaggregate activities by type. Finally, in the rightmost column of Table 3 we report the share of total person-days on farm worked by hired laborers. The percentages are surprisingly consistent across countries, with the average slightly above 10% and only Ethiopian households demonstrating significantly greater usage of hired workers. The rightmost column of Table 3 shows the average payment per hired work-day, by activity and country. These figures are not exact measures of daily wages, because they are derived from aggregate data. There are few important patterns to the payments data, though two noteworthy points are that the average payment in Uganda is much lower than elsewhere, and that the harvest wage is the lowest or approximate lowest payment in three of the other four countries.

The clear message of Table 2, Table 3 is that a large share of African agricultural households hire in labor, land, or both, in any given year. Even excluding harvest labor hiring and those households that hire themselves out as agricultural workers and do not hire others, at least a large minority of cultivating households participate in labor or land markets, or both. Clearly these markets exist and have sufficient numbers of transactions that households generally act as price-takers. But the existence of markets with adequate transactional density is merely a necessary condition for the separation hypothesis to hold. As de Janvry et al. (1991) make clear, markets can fail idiosyncratically for specific households for any of a host of reasons. Furthermore, if other rural markets (output, insurance, credit) are missing, this can manifest as a violation of separation in the labor demand equations.

In Table 4 we document patterns of household participation in markets or programs related to output, insurance, and credit. The percentage of households that report selling output ranges from 28.3% in Niger to 62.9% in Ethiopia, though these estimates are surely lower bounds on output market participation as they ignore any sales from stocks that take place in the months between the survey and the next harvest. A smaller percentage of households report borrowing from financial institutions, SACCOs, or other households, with rates ranging from 13.3% in Malawi and Tanzania to 40.8% in Uganda. Between 16.3% (Ethiopia) and 56.4% (Niger) of households report receiving a transfer from another household, a form of social insurance that acts as a *de facto* form of partial crop insurance in agricultural communities. Overall it is clear that these markets, like those for land and labor, are in widespread operation across the study countries. Of course, it remains highly likely that some households are crowded out of some markets, especially those for credit or certain transfers, because of risk-adjusted pricing or rationing by local institutions with significant information about potential participants.

**Fig. 1:**
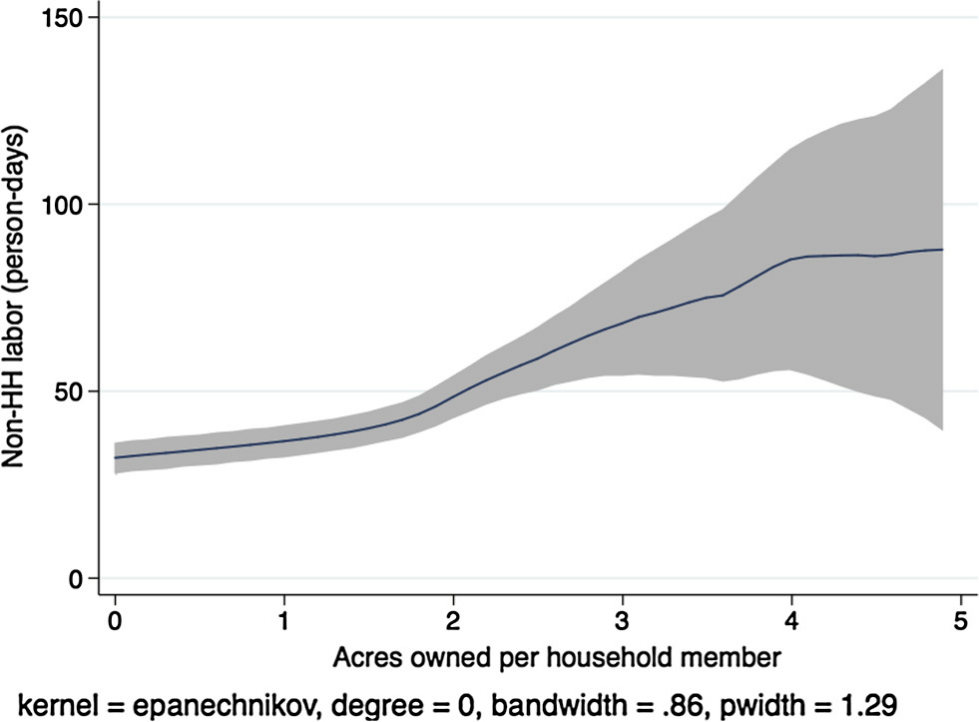
Kernel regression, non-household labor on land:labor endowment ratio, Ethiopia.

Next, descriptive kernel regressions of labor hiring and labor demand patterns give a preview of the multivariate regression results we present in the next section, where we test the separation hypothesis formally. Fig. 1 shows a local polynomial regression at the household level of the demand for non-household hired labor (person-days) on the household land-to-labor endowment ratio (acres owned per household member) in Ethiopia.^10^ In aggregating labor across individuals, we count each child person-day as 50% of an adult person-day. Grey shading indicates the 95% confidence interval around the regression estimate.

There are two things to note in the figure. First, labor hiring is statistically significantly increasing in the number of acres owned per household member, as we would expect in a country with at least some agricultural labor market activity. Second, to the extent that any patterns are apparent in the figure, the relationship is concave at higher levels of owned acreage. Or, if linear, the slope of the regression line is clearly less than one. As the number of acres per household member increases, hiring of outside workers does not increase proportionally. In theory, this could be due to economies of scale in either household labor, hired labor, or both, although the empirical literature on smallholder agricultural production routinely supports the constant returns to scale hypothesis. However, this pattern is also consistent with labor or credit market failures that prevent households with greater need for outside laborers from hiring at optimal levels.

**Fig. 2:**
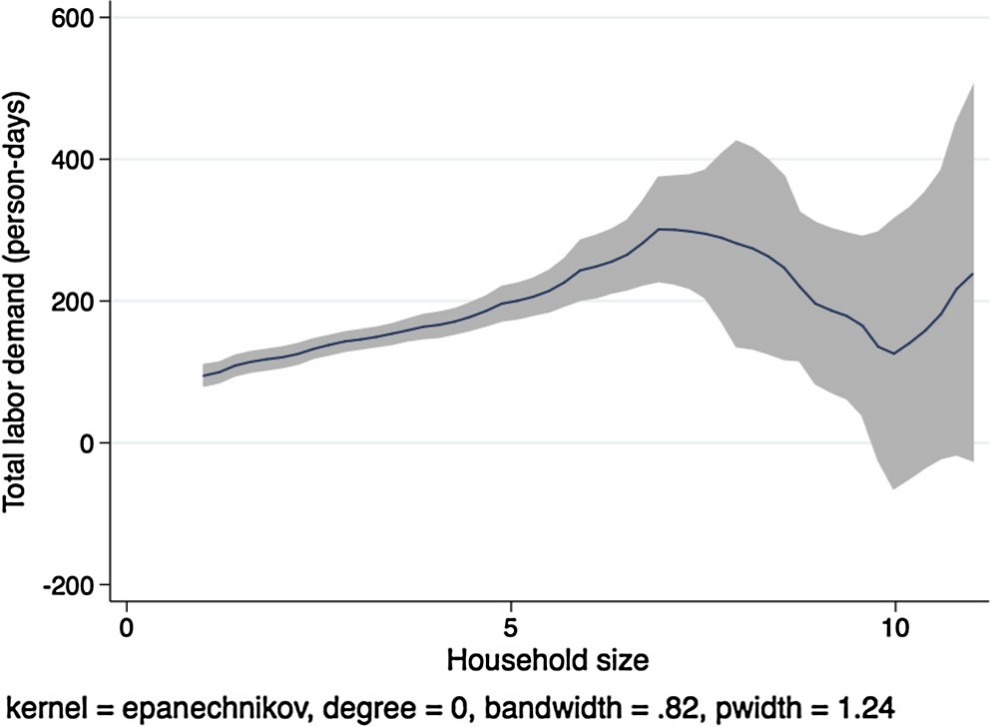
Kernel regression, total labor demand on household size, Ethiopia.

Fig. 2 shows a kernel regression of total labor demand (family labor plus hired labor, in adult person-days) on total household size, again for households in Ethiopia. If labor markets were complete and competitive and the separation hypothesis held, we would expect to see no clear relationship between these two variables. Instead we see that total labor demand is increasing in the number of household members until a household size of 7, after which the regression line tapers off and becomes noisier. Although this figure does not constitute a formal separation test, because the underlying result does not condition on important covariates, it does suggest that there exists a strong relationship between household labor endowments and the application of labor on the family farm.

**Fig. 3:**
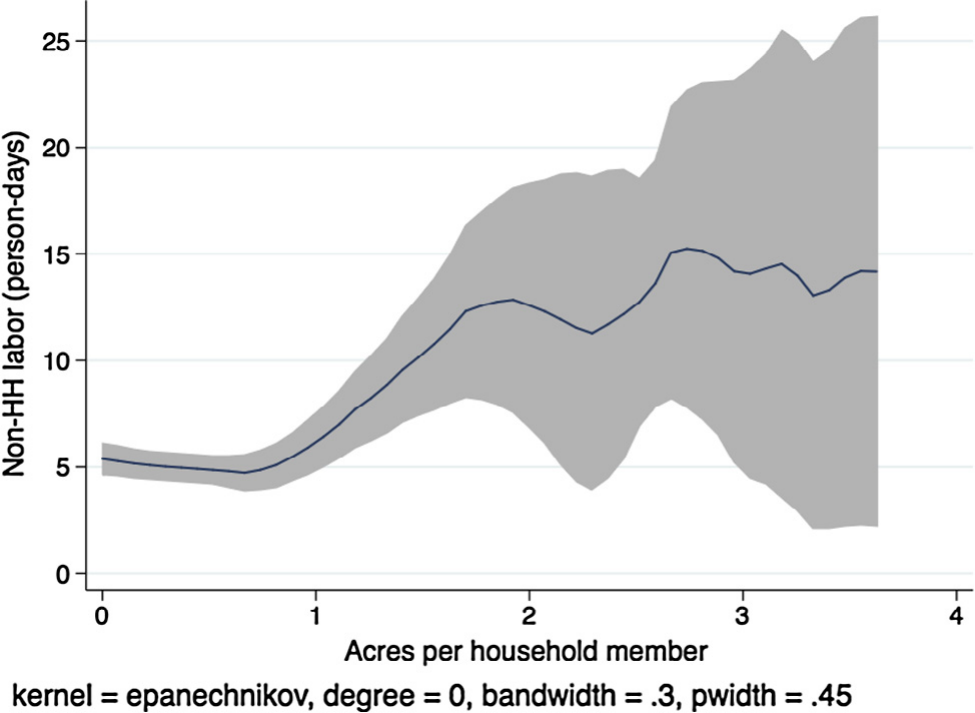
Kernel regression, non-household labor on land:labor endowment ratio, Malawi.

**Fig. 4:**
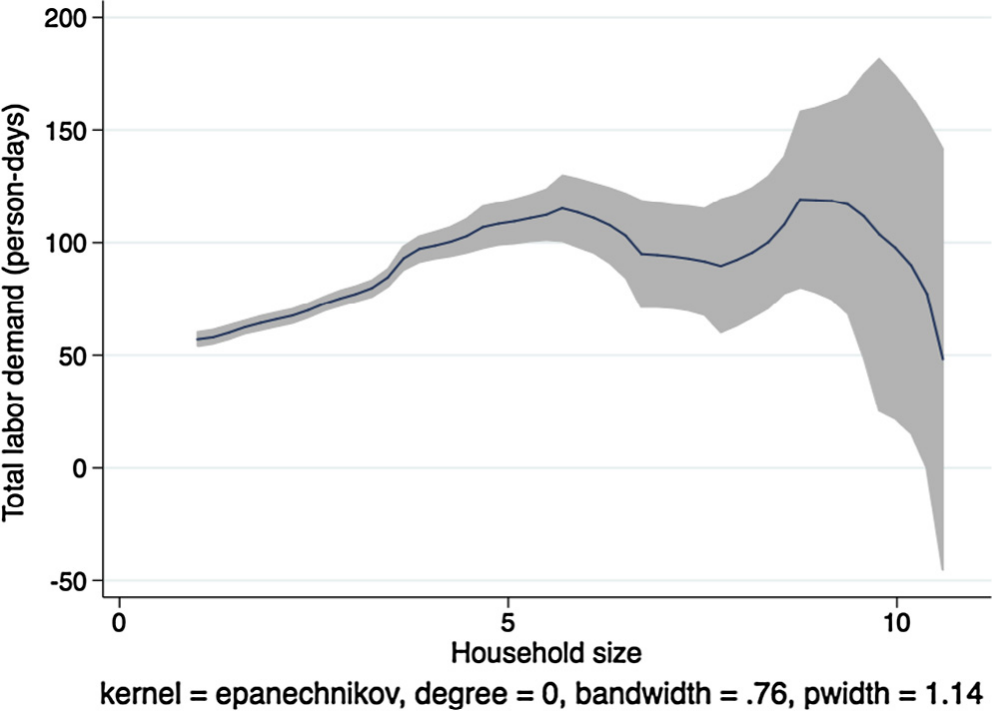
Kernel regression, total labor demand on household size, Malawi.

**Fig. 5:**
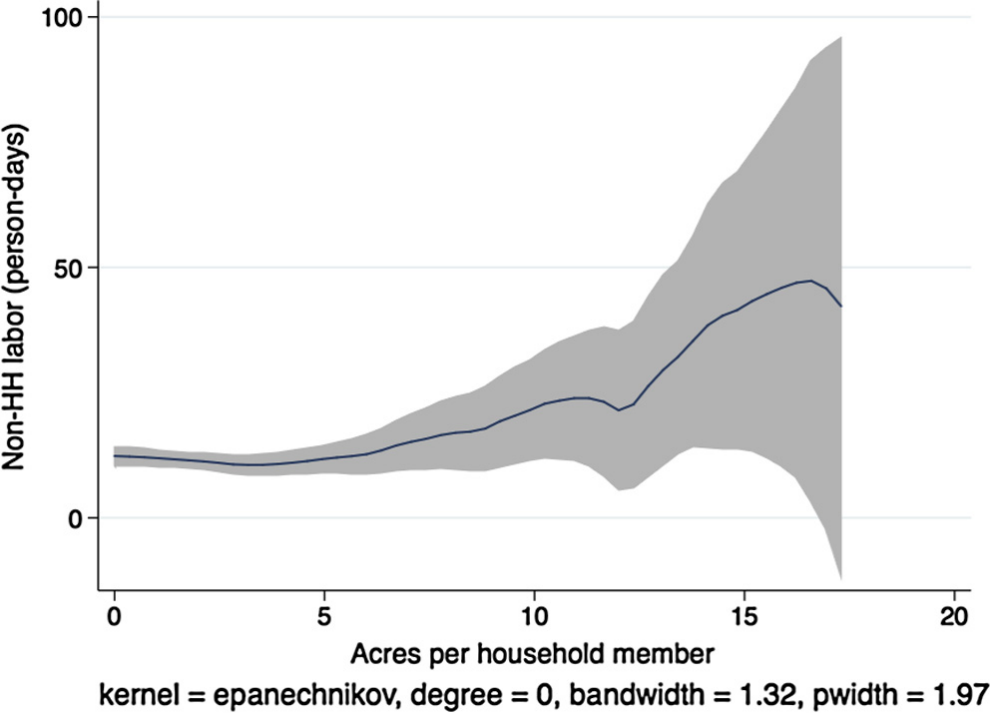
Kernel regression, non-household labor on land:labor endowment ratio, Niger.

**Fig. 6:**
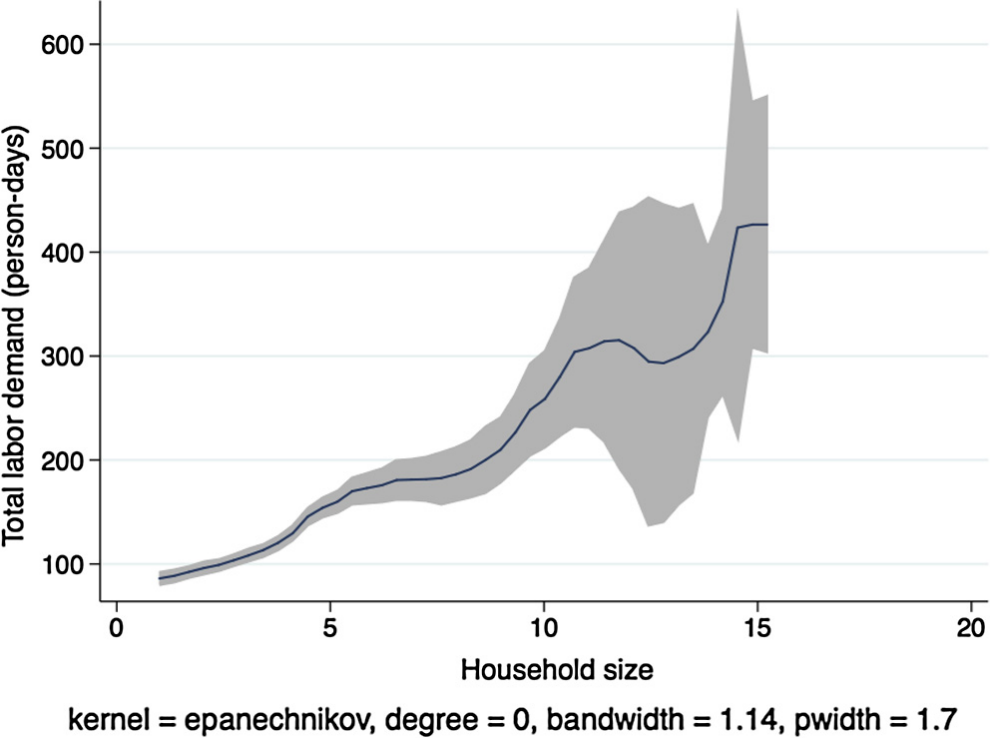
Kernel regression, total labor demand on household size, Niger.

**Fig. 7:**
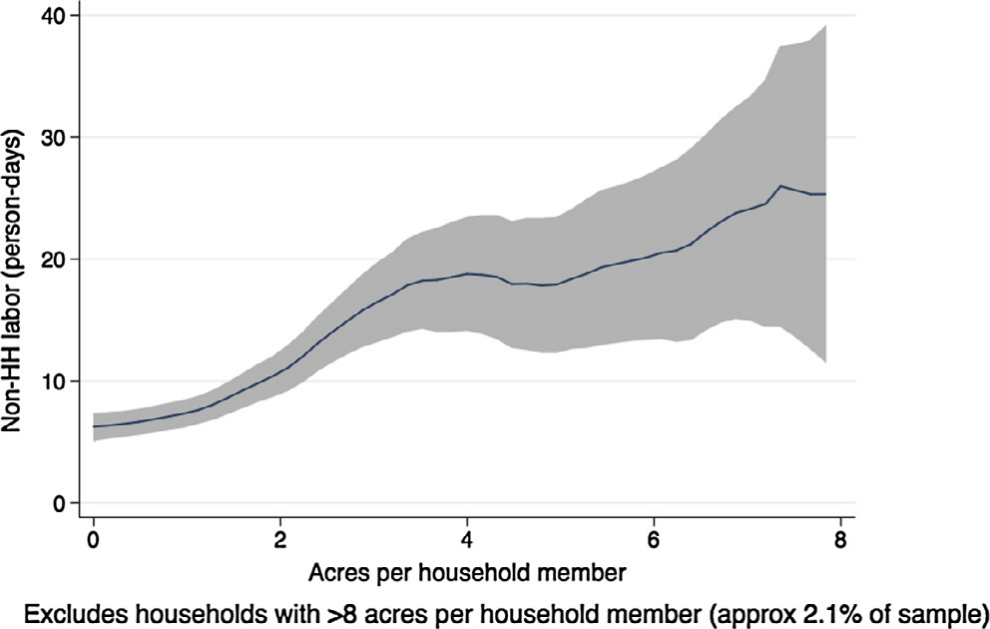
Kernel regression, non-household labor on land:labor endowment ratio, Tanzania.

**Fig. 8:**
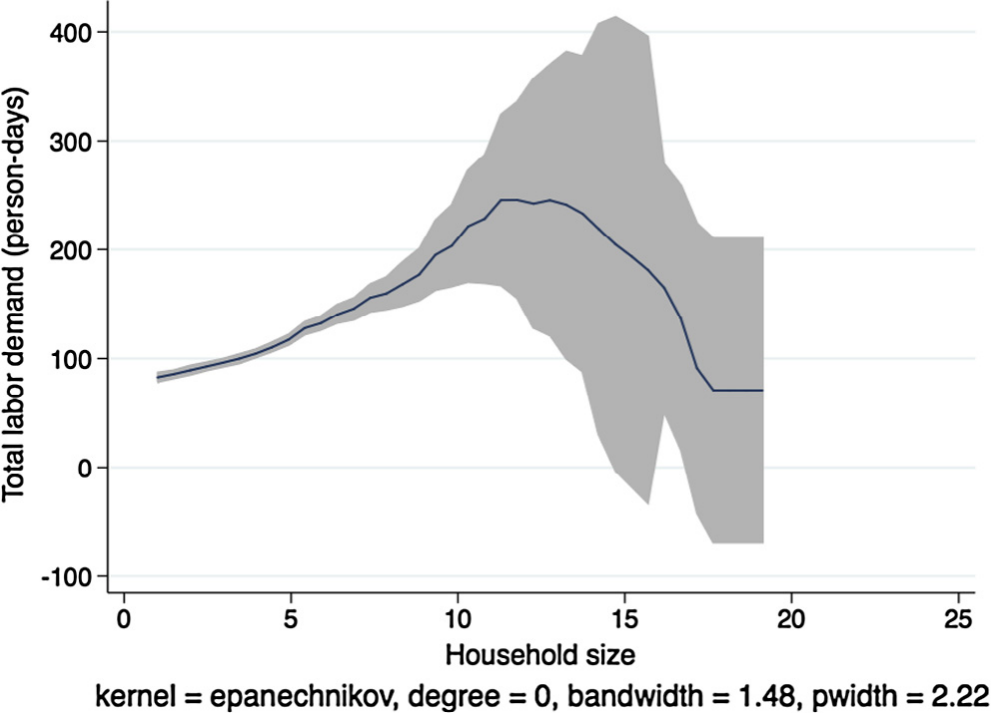
Kernel regression, total labor demand on household size, Tanzania.

**Fig. 9:**
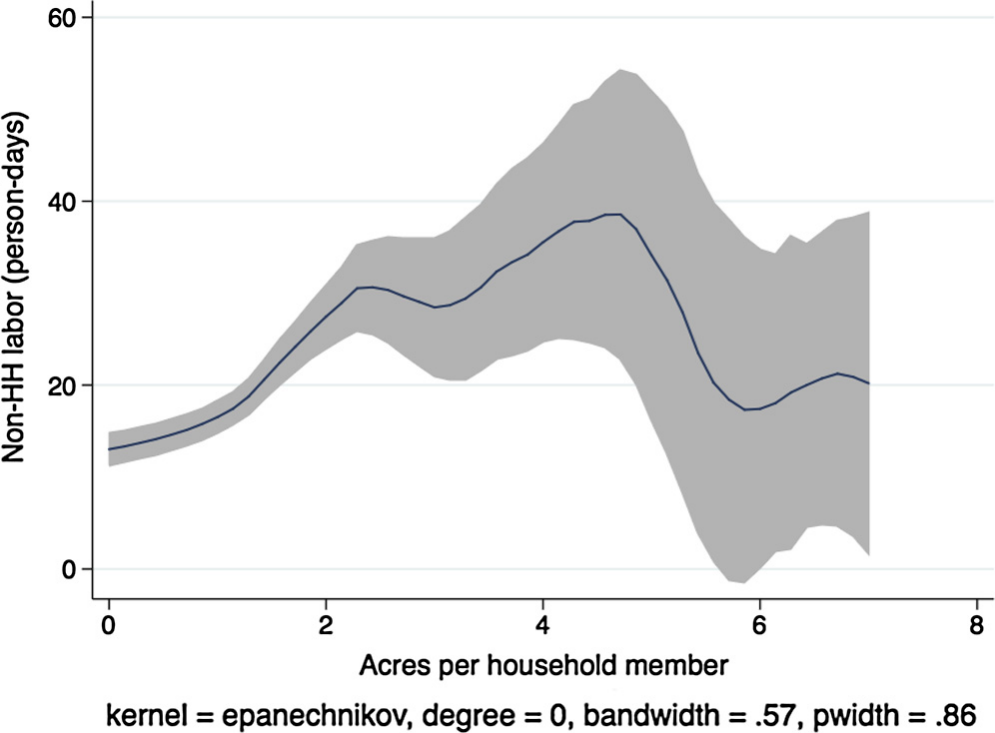
Kernel regression, non-household labor on land:labor endowment ratio, Uganda.

**Fig. 10:**
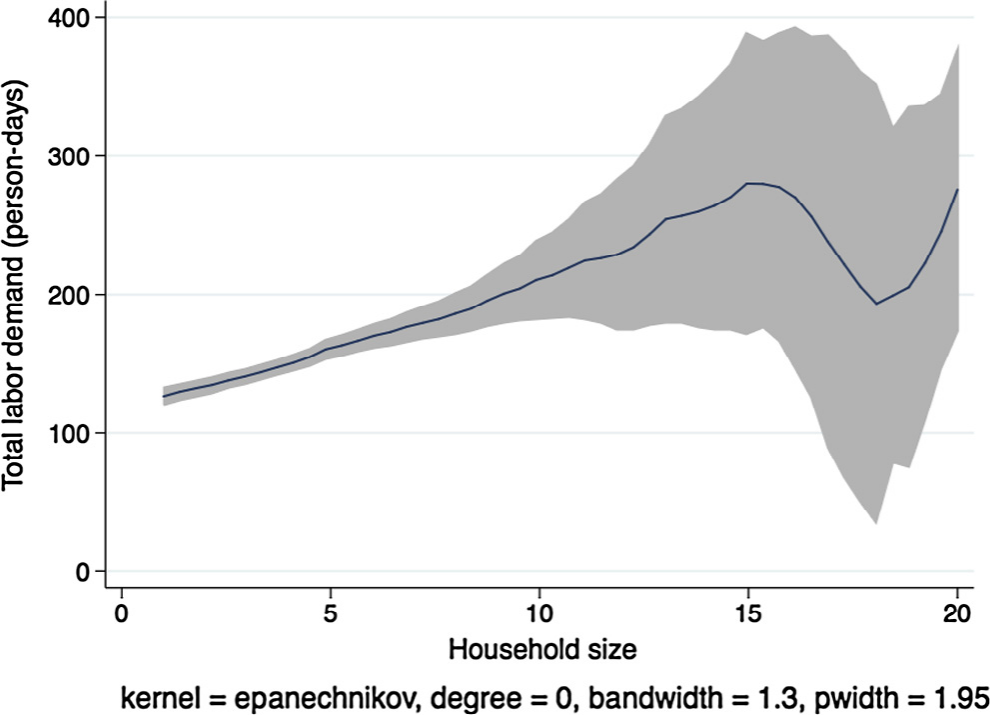
Kernel regression, total labor demand on household size, Uganda.

Fig. 3, Fig. 4, Fig. 5, Fig. 6, Fig. 7, Fig. 8, Fig. 9, Fig. 10 show similar pairs of kernel regression results for Malawi, Niger, Tanzania, and Uganda, respectively. The general patterns in Fig. 3, Fig. 7, Fig. 9 are consistent with that from Fig. 1 for Ethiopia: hiring of outside workers is increasing in the number of acres per household member, but at a decreasing marginal rate. In Fig. 5, however, we see no such pattern for households in Niger, particularly in the region over which most of the data are concentrated, i.e., below eight acres per person. Instead, the number of person-days demanded from outside workers is flat or even slightly decreasing in the household land:labor endowment ratio. One interpretation of this result, in combination with the observation that households in Niger rent significantly more land than those in other study countries, is that credit-constrained households borrow against expected harvest output to rent land, but that such loans cannot be converted into cash to hire workers.

In Fig. 4, Fig. 6, Fig. 8, Fig. 10, the picture is clearer and absolutely consistent with that of Fig. 2. Total labor demand is increasing in household size in all study countries. This pattern anticipates the formal hypothesis testing results presented below, that household factor demand varies strongly with household labor endowments, contradicting the separation hypothesis implied by the canonical agricultural household model under the assumption of complete and competitive factor markets.

## 4 Regression results

In Table 5 we show summary statistics for the variables used in the country-specific regression estimates of Eq. (12). Median wages in local currency units are based on reported wages, including both cash and in-kind payments. These median wages are calculated at the smallest level of geographical aggregation with at least 10 observations (beginning at the zone, *grappe*, or TA level, depending on the country, and moving to larger areas as needed). “Prime age” is defined as ages 15–60 years, while “Elderly” indicates age >60 years. The excluded demographic category is elderly male. Note that the household size variable used here is the sum of those two age categories; we omit children under age 15 so as to mitigate concerns about the possible endogeneity of household size and so as not to mix adults with young children unable or unlikely to work. Note that we do, however, still count child contributions to agricultural labor demand, assuming that one child work day is equivalent to half an adult’s work day.

The results of the basic ordinary least squares (OLS) implementation of the separation hypothesis test from Eq. (12) are reported in Table 6. All regressions are weighted by sampling probabilities, and standard errors are clustered at the level of the zone (Ethiopia), TA (Malawi), *grappe* (Niger) or district (Uganda and Tanzania). We include location fixed effects in all models, to control for spatial variation in prices. All of the signs of the estimated coefficients in Table 6 are consistent with expectations, when statistically significant. The estimated elasticity of labor demand with respect to acres owned ranges from 0.08 in Niger to 0.19 in Ethiopia and is statistically significant at the one percent level in all cases. The household composition variables are for the most part not statistically significant, although when they are we see that labor demand is increasing in the share of prime age adults and decreasing in the share of elderly females (relative to elderly males). Finally, and most importantly for this paper, the null hypothesis of separation can be strongly rejected at the 1 percent level of significance in all regressions in Table 6. The estimated elasticity of labor demand with respect to household size (i.e., number of adults) ranges from 0.32 in Uganda to 0.75 in Niger. The magnitude of this elasticity can be taken as a rough indicator of the depth of market failure. In this sense the findings are similar to those from the kernel regressions, in that demand side participation in labor markets appears weaker in Niger than in the other study countries. Although many households in Niger hire agricultural laborers (Table 3), the total amount of labor applied to farms in Niger is linked more closely to the (larger) size of Nigerien households than it is in the other study countries. Overall, the consistent message in Table 6 is that agricultural households in all study countries are not served by complete and competitive markets. Market failures create a dependency on endowments.

### 4.1 Are there patterns in market failures?

The preceding results describe a generalized, structural failure of multiple factor markets in rural Africa. But are these problems perhaps especially concentrated among identifiable subpopulations? In order to explore that question we examine some of the household- and location-level correlates of factor market failure. Our approach remains strictly reduced form. In order to test whether a particular characteristic is associated with a greater or lesser degree of market failure, we include the variable in Eq. (12) both independently and interacted with the log of household size variable. We are especially interested to see if these new variables diminish the magnitude or eliminate the statistical significance of the estimated relationship between log household size and labor demand. Such a result would suggest that factor market failures affect primarily distinct subpopulations and are not generalized within rural Africa. We consider three primary sources of heterogeneity in access to complete markets: gender of the household head, distance from key points such as paved roads and large population centers, and agro-ecological zone. We conclude with a small number of additional robustness checks to control for variation in wealth, human capital, and soil quality.

Gender inequities in access to credit, inputs, labor, markets, public services and technologies are often presumed to be widespread in rural Africa. There is indeed significant prior evidence of productivity differences by gender (Udry, 1996, Doss and Morris, 2000). Likewise, greater distances from paved roads and trading centers may proxy for prohibitively high transaction costs, which prior studies have shown are correlated with agricultural productivity (Stifel and Minten, 2008). Sub-optimal investment in infrastructure can lead to incomplete factor markets, for example through stock-outs of seeds and fertilizer (because of prohibitively high transaction costs) or thin labor markets. Lastly, we consider whether variation in agro-ecological zone, which serves as a proxy for agricultural potential, explains variation in the degree of market failure.

Table 7 shows the results of regressions with controls for the gender of the household head. Perhaps surprisingly, we find little evidence of heterogeneity in factor market performance by gender of the head. In all study countries other than Niger, the coefficients on both the level and interaction variables are statistically insignificant and of relatively small magnitude. In Niger, the elasticity of labor demand with respect to household size is smaller by about half for female-headed households than for male-headed households. The differences by gender are not of inconsequential magnitudes. Yet, as indicated in the last row of the table, we can reject separation for female-headed households in Niger (p-value = 0.06). The small and statistically insignificant coefficients on the interaction terms between log household size and female headedness indicate that there are no meaningful differences between male- and female-headed households in Ethiopia, Malawi, Tanzania, and Uganda. F-tests confirm that for all five countries we can reject the null hypothesis that the effect of log household size on log labor demand is zero for female-headed households (and clearly the same is true for male-headed households). Overall, it does not appear that gender of the household head helps in explaining variation in the completeness of the markets facing rural households.

Distance variables are similarly uninformative. For each study country we estimated Eq. (12) including interactions and levels for four different distance variables (separately): distance from a paved road, distance from the closest town with 20,000+ inhabitants, distance from a large market,^11^ and distance from the region or district capital. Regression coefficients on distance variables are of negligible magnitude, and in no cases are the results of statistical and economic significance. Additionally, inclusion of distance controls does not have a significant effect on the coefficient estimate on household size, so that the estimated violation of the separation hypothesis is not attenuated in any of the samples. These results are shown in Table 8. While it might be reasonable to conjecture that rural market failures would be most acutely felt in more remote areas, we find no evidence of such a pattern by any of several common measures of market access.

Next we examine whether the degree of market failure varies with the agro-climate (Binswanger and Rosenzweig, 1986). For each country we include in Eq. (12) a set of dummy variables and interactions with log household size for the relevant agro-ecological zones (AEZs). In each case we exclude the most common AEZ (at the country level). Because each country has only a small number of spatially correlated AEZs, we omit the location fixed effects and instead use locally estimated median wages to control for spatial price variation.

To aid in interpretation, Table 9 shows the distribution of AEZs across households in each country sample, as well as the mean and standard deviation of the log HH size variable. In most of the well-represented AEZs the mean of “Log HH size” is slightly greater than one.

Table 10 reports the regression results. In Malawi, Niger, and Uganda there are no significant differences between AEZs in the way that household labor endowments relate to labor demand. In Malawi the baseline effect for the omitted AEZ is positive and highly significant, and the interaction terms between household size and other AEZs are all positive and not different from zero (so the net effect in all AEZs is positive). In Niger there is a significant level difference in conditional labor demand between AEZs, with greater demand in arid areas than semi-arid areas, but the interaction with log of household size is again not significant. In Uganda the estimated coefficients on interaction terms between AEZs and household size are very small and statistically indistinguishable from zero. Given the nearly uniform insignificance of the AEZ interaction terms in Table 10, it is not surprising that the baseline “Log HH size” effects for these three countries are close to the values reported in Table 6 for the parsimonious specification.

For Ethiopia, the only statistically significant difference in Table 10 (from the baseline category of cool, sub-humid tropics) is in the warm arid areas (T, W, A), where the net effect of household size on labor demand is not statistically different from zero.^12^ As we see in Table 9, however, this result is based on only 34 households (the areas of Ethiopia classified as warm and arid are in relatively sparsely populated parts of the Afar and Somali regions).

Only in Tanzania is there any indication that the degree of rural market failures might vary between agro-ecological zones. In column 4 of Table 10 we see that interaction terms between household size and AEZ dummy variables are statistically different from zero, suggesting that the relationship between household size and labor demand differs from that of the excluded AEZ (warm sub-humid tropics). All of the interaction terms are positive, indicating that in all cases, conditional labor demand increases much more quickly with household size in the included AEZs than it does in the warm sub-humid tropics. Differences in the elasticity of labor demand with respect to household size range from 0.21 in the cool sub-humid tropics to 0.70 in the cool humid tropics. This is suggestive evidence that within Tanzania, factor market failures are greatest in areas outside of the warm sub-humid tropics. This is not surprising, because the warm sub-humid tropics are home to the bulk of cultivation in Tanzania. It appears that rural market failures are most acute where agricultural production is least concentrated.

Finally, we consider numerous other potential sources of heterogeneous effects or confounding variation from variables that may influence both household size and labor demand on farm. First we split the sample into households above and below median expenditure per capita, because wealthier households may have greater capacity to hire labor when needed or to acquire additional land when endowed with enough labor to farm it. Second, in a separate set of regressions we control for the education level of the household head, as well as interactions between the head’s education and the log of household size. Here, education proxies for overall human capital. Third, because it remains possible that household size is endogenous to unobserved farm quality, we included additional controls for the share of owned land of various soil types (sandy, loam, clay). Data on soil type is not available for Ethiopia. Table 11 shows the main results from each of these additional tests (full results available upon request). In all cases, the relationship between household size and labor demand on farm remains economically and statistically significant. The level values of the estimated elasticities are essentially unchanged from those reported in the main tables.

## 5 Conclusions

Using a theoretically-grounded, reduced form test for complete and competitive markets, we have shown that the relationship between labor demand and household size is broadly consistent with the existence of widespread, multiple market failures across agrarian communities in five Sub-Saharan African countries. Despite widespread participation in labor and land markets by agricultural households across SSA, regression results based on a simple, reduced form exclusionary restriction derived from the first order necessary conditions of the household optimization problem indicate that household endowments influence factor demand in a way that is inconsistent with the separation hypothesis implied by the assumption of complete and competitive factor markets. This finding corresponds with the unconditional results suggested by simple kernel regressions of labor demand on household factor endowments. The overall conclusion supports the widespread sense among the development community that rural markets regularly fail African farmers. But it is important to note that the tests implemented here, even though they rely on an analysis of labor market transactions, do not allow us to identify precisely *which* factor markets fail, as violations of the separation hypothesis can occur even with perfectly functioning labor markets (Barrett, 1996). The fact that a large share of agricultural households transact in rural labor, land, and output markets suggests that the issue is less one of outright market absence than of structural barriers – perhaps related to financial intermediation, uncertain and expensive contract enforcement, weak physical infrastructure that results in high transactions costs, etc. – that impede efficient factor market functioning for most rural households. The fact that these market failures are not concentrated among households readily identified by location or gender signals that these market failures are general and structural.

As the development community and African governments increasingly intervene to try to rectify perceived market failures, the onus now falls on researchers to more precisely locate the sources and causes of factor market failures that impede productivity and income growth in rural Africa. Effective targeting of interventions depends on more precise, structural estimation that goes beyond the reduced form tests we offer in this paper. This will require methodological advances to take advantage of data now becoming available to help inform the design and evaluation of policies intended to help stimulate African agricultural and rural development.

## Figures and Tables

**Table 1:** Household-level summary statistics for all study countries.

	Ethiopia			Malawi			Niger			Tanzania			Uganda		
	Mean	s.d.	N	Mean	s.d.	N	Mean	s.d.	N	Mean	s.d.	N	Mean	s.d.	N
Household size	5.24	2.2	3094	4.96	2.29	2666	6.78	3.53	2339	5.55	2.95	2630	6.64	3.46	2135
Male head = 1	0.81		3094	0.77		2666	0.92		2339	0.75		2630	0.71		2135
Education of head (yrs)	1.61	2.72	3039	5.22	4.18	2646	0.87	2.35	2339	4.58	3.37	2582	4.69	3.34	1813
Number of males	2.66	1.52	3094	2.4	1.53	2666	3.37	2.09	2339	2.7	1.8	2630	3.22	2.09	2135
Number of females	2.56	1.38	3094	2.56	1.44	2666	3.41	2.11	2339	2.85	1.78	2630	3.43	2.11	2135
No. plots own/cultivate	11.69	7.33	3094	2.05	1.13	2666	3.04	1.96	2339	2.3	1.32	2630	5.65	2.69	2135
Acres owned	2.81	5.25	3094	1.58	1.73	2666	11.98	19.65	2339	5.31	10.51	2630	3.29	10.27	2135
Share cultivate	0.76	0.26	2790	0.99	0.1	2043	0.98	0.12	1969	0.81	0.34	2341	0.9	0.24	1952
Share rent out				0.01	0.07	2043	0.01	0.08	1969	0.02	0.12	2341	0	0.05	1952
Share fallow	0.03	0.11	2790	0.01	0.06	2043	0.01	0.07	1969	0.15	0.31	2341	0.07	0.2	1952
Share other	0.22	0.24	2790	0	0.01	2043	0.01	0.07	1969	0.03	0.14	2341	0.02	0.14	1952
Acres cultivated	2.7	4.23	3094	1.92	1.73	2666	13.62	19.05	2339	4.31	7.93	2630	2.98	7.41	2135
Share own	0.82	0.32	2810	0.79	0.39	2588	0.81	0.35	2202	0.86	0.32	2409	0.79	0.34	2073
Share rent/borrow	0.18	0.32	2810	0.21	0.39	2588	0.19	0.35	2202	0.14	0.32	2409	0.21	0.34	2073
Maize: acreage	0.49	1.83	2826	1.45	1.27	2567				2.2	4.13	2402	0.41	2.21	2075
Sorghum: acreage	0.37	1.12	2826				2.42	4.49	2127						
Rice: acreage				0.03	0.23	2567				0.44	2.13	2402	0.03	0.21	2075
Millet: acreage							6.14	8.25	2127						
Other grains: acreage	1.01	1.54	2826	0.03	0.24	2567	0.13	0.74	2127	0.39	2.45	2402	0.21	0.88	2075
Tubers: acreage	0.02	0.09	2826	0.04	0.31	2567	0.05	0.41	2127	0.57	2.11	2402	0.94	5.14	2075
Pulses/nuts: acreage	0.32	0.88	2826	0.22	0.51	2567	4.56	6.44	2127	0.49	5.18	2402	0.62	2.47	2075
Cash crops: acreage	0.24	0.79	2826	0.18	0.57	2567	0	0.06	2127	0.34	2.25	2402	0.23	1.47	2075
Other crops: acreage	0.23	0.55	2826	0.01	0.1	2567	0.37	1.85	2127	0.31	1.83	2402	0.61	3.29	2075

**Table 2:** Participation in land rental markets.

	Ethiopia^a^	Malawi	Niger	Tanzania	Uganda
N	3094	2666	2339	2630	2135
Household rents land out	7.4%	0.7%	1.2%	3.7%	0.4%
Household rents land in	22.2%	11.9%	8.5%	7.5%	20.1%
Household rents or borrows land in	32.7%	24.9%	30.9%	19.6%	38.7%
Acres rented out (mean)	0.574	0.015	0.094	0.169	0.006
Acres rented out (sd)	1.455	0.204	1.044	2.177	0.105
Acres rented in (mean)	0.605	0.381	2.156	0.404	0.615
Acres rented in (sd)	1.393	0.988	6.254	1.294	3.020

a Acres rented out is not identifiable in the ISA Ethiopia data; reported statistics are for “other” uses of land, which appears to be an upper bound on land rentals; summary statistics based on survey weights.

**Table 3:** Percent of agricultural households hiring labor (weighted).

Country	Activity	Number of households	Percent hiring workers	Percent of work done by hired labor	Average payment per day to hired workers (USD)
Ethiopia	Cultivation	3091	18.5%	16.0%	2.96
	Harvest	2666	20.9%	20.1%	3.87
	Overall	2666	30.2%	17.3%	

Malawi	Non-harvest	2605	30.7%	9.2%	4.05
	Harvest	2605	16.0%	6.7%	2.70
	Overall	2605	40.1%	8.6%	

Niger	Preparation	2339	18.2%	8.8%	4.42
	Cultivation	2339	38.2%	10.5%	3.88
	Harvest	2339	19.0%	8.5%	2.53
	Overall	2339	48.7%	9.7%	

Tanzania	Planting	2630	18.1%	8.3%	3.47
	Weeding	2630	19.6%	9.9%	2.34
	Fertilizing	2630	2.1%	4.8%	2.76
	Harvest	2630	15.8%	8.2%	2.41
	Overall	2630	30.1%	8.8%	

Uganda	Overall	2109	45.2%	10.0%	1.35

*Notes:* Authors’ calculations from LSMS-ISA data; some variables collected as average hours per day, we convert to full days using conversion rate 6 h = a full work day; average payment calculated by adding all days worked by adult men and women, plus 0.5 days per child day, and dividing by total payments to workers for the listed activity, including in-kind payments; exchange rates to convert to USD are from July 2009 (Malawi), July 2010 (Niger, Tanzania, Uganda), July 2011 (Ethiopia).

**Table 4:** Rates of household participation in markets and programs.

	Ethiopia	Malawi	Niger	Tanzania	Uganda
Sold any output	62.9%	39.5%	28.3%	49.2%	49.2%
Took out loan/accessed credit	27.5%	13.3%		13.3%	40.8%
Received transfer from NGO or Govt. program	13.7%	17.9%		35.6%	
Received transfer from other household	16.3%	31.0%	56.4%		27.6%

At least one of the above	80.0%	68.8%	67.3%	69.0%	78.2%

*Notes:* Output sales include sales of tree crops but not sales from stocks that occur post-survey; credit access includes loans from SACCOs or other households, when specified; reported averages are weighted; missing values are absent because question not included in survey.

**Table 5:** Summary statistics of main variables used in regressions.

	Ethiopia	Malawi	Niger	Tanzania	Uganda
Log labor demand (person-days)	4.47	3.85	4.26	4.27	4.72
	1.23	0.97	0.98	0.98	0.77
Log acres owned	0.10	−0.52	1.36	0.71	0.19
	1.78	2.04	2.39	1.89	1.79
Log HH size	1.17	0.84	1.02	0.98	1.12
	0.44	0.45	0.46	0.49	0.58
Log median wage (local currency)	2.768	5.563	6.998	7.82	8.761
	1.083	0.539	0.443	0.489	0.649
Prime male share	0.33	0.40	0.42	0.40	0.37
	0.20	0.23	0.18	0.24	0.23
Prime female share	0.37	0.48	0.51	0.46	0.43
	0.20	0.24	0.16	0.24	0.24
Elderly female share	0.14	0.07	0.03	0.08	0.11
	0.20	0.21	0.11	0.20	0.21

N	2499	2556	2183	2346	2047

*Notes:* Authors’ calculations from LSMS-ISA data sets; main entries are mean values, with standard deviations below; survey weights used.

**Table 6:** Regression results from parsimonious OLS specification w/district FE.

	Ethiopia	Malawi	Niger	Tanzania	Uganda
*Dependent variable: Log labor demand*					
Log acres owned	0.186⁎⁎⁎	0.098⁎⁎⁎	0.083⁎⁎⁎	0.129⁎⁎⁎	0.141⁎⁎⁎
	(0.027)	(0.012)	(0.011)	(0.016)	(0.014)
Log HH size	0.616⁎⁎⁎	0.667⁎⁎⁎	0.747⁎⁎⁎	0.616⁎⁎⁎	0.315⁎⁎⁎
	(0.053)	(0.048)	(0.076)	(0.048)	(0.039)
Prime male share	0.734⁎⁎⁎	−0.11	0.038	−0.036	0.348⁎⁎
	(0.129)	(0.129)	(0.243)	(0.147)	(0.145)
Prime female share	0.095	−0.251	−0.263	−0.204	0.331⁎⁎
	(0.221)	(0.152)	(0.243)	(0.147)	(0.156)
Elderly female share	−0.389⁎⁎	−0.12	−0.891⁎⁎⁎	−0.348⁎	−0.039
	(0.161)	(0.197)	(0.310)	(0.202)	(0.162)
Constant	3.397⁎⁎⁎	3.713⁎⁎⁎	4.450⁎⁎⁎	4.052⁎⁎⁎	3.186⁎⁎⁎
	(0.147)	(0.127)	(0.273)	(0.121)	(0.146)
District/zone FE	Yes	Yes	Yes	Yes	Yes

R-squared	0.355	0.343	0.452	0.364	0.313

N	2765	2556	2183	2364	2047

*Notes:* Standard errors in parentheses; standard errors clustered at the level of the zone (Ethiopia), TA (Malawi), grappe (Niger) or district (Tanzania and Uganda); fixed effects included at those same geographical levels; sampling weights used for all regressions; dependent variable is the log of total labor demand, defined as total person-days employed on all plots; children under age 15 are counted as 0.5 adults; harvest labor is excluded for ET, MW, NG, and TZ, but included for UG because it cannot be separately distinguished; population shares defined with respect to adults > age 14.

⁎⁎⁎ Significant at 1%.

⁎⁎ Significant at 5%.

⁎ Significant at 10%.

**Table 7:** OLS results with controls for gender of household head.

	Ethiopia	Malawi	Niger	Tanzania	Uganda
*Dependent variable: Log labor demand*					
Log acres owned	0.187⁎⁎⁎	0.097⁎⁎⁎	0.083⁎⁎⁎	0.128⁎⁎⁎	0.140⁎⁎⁎
	(0.027)	(0.012)	(0.011)	(0.016)	(0.014)
Log HH size [A]	0.579⁎⁎⁎	0.680⁎⁎⁎	0.816⁎⁎⁎	0.588⁎⁎⁎	0.331⁎⁎⁎
	(0.085)	(0.073)	(0.074)	(0.061)	(0.049)
Prime male share	0.723⁎⁎⁎	−0.104	0.048	−0.002	0.346⁎⁎
	(0.133)	(0.130)	(0.249)	(0.153)	(0.149)
Prime female share	0.2	−0.202	−0.461	−0.079	0.326⁎⁎
	(0.203)	(0.174)	(0.282)	(0.189)	(0.149)
Elderly female share	−0.307⁎	−0.054	−1.219⁎⁎⁎	−0.175	−0.043
	(0.155)	(0.232)	(0.323)	(0.254)	(0.159)
Head is female (=1)	−0.138	−0.018	0.450⁎⁎	−0.149	0.031
	(0.179)	(0.145)	(0.199)	(0.130)	(0.084)
Head is female (=1) × Log HH size [B]	−0.077	−0.063	−0.470⁎⁎⁎	0.081	−0.047
	(0.155)	(0.139)	(0.179)	(0.113)	(0.061)
Constant	3.441⁎⁎⁎	3.680⁎⁎⁎	4.472⁎⁎⁎	4.020⁎⁎⁎	3.179⁎⁎⁎
	(0.175)	(0.136)	(0.289)	(0.130)	(0.152)

District/zone/cluster FE	Yes	Yes	Yes	Yes	Yes

R-squared	0.36	0.34	0.46	0.36	0.31
N	2765	2556	2183	2364	2047
F-test: [A] + [B] = 0, test statistic	19.66	41.58	3.62	56.42	32.88
F-test: [A] + [B] = 0, p-value	0.00	0.00	0.06	0.00	0.00

*Notes:* Standard errors in parentheses; standard errors clustered at the level of the zone (Ethiopia), TA (Malawi), grappe (Niger) or district (Tanzania and Uganda); sampling weights used for all regressions; dependent variable is the log of total labor demand, defined as total person-days employed on all plots; children under age 15 are counted as 0.5 adults; harvest labor is excluded for ET, MW, NG, and TZ, but included for UG because it cannot be separately distinguished; population shares defined with respect to adults > age 14; for households with zero acres owned, “Log acres owned” = ln(0.01).

⁎⁎⁎ Significant at 1%.

⁎⁎ Significant at 5%.

⁎ Significant at 10%.

**Table 8:** OLS results including interactions with distance to key points.

	Ethiopia	Malawi	Niger	Tanzania	Uganda
*Dependent variable: Log labor demand*					
Log HH size	0.592⁎⁎⁎	0.719⁎⁎⁎	0.717⁎⁎⁎	0.516⁎⁎⁎	0.225⁎⁎⁎
	(0.099)	(0.101)	(0.125)	(0.067)	(0.061)
Distance to large town (km)	−0.001	0.007⁎	−0.015	0.001	0.001
	(0.004)	(0.004)	(0.013)	(0.002)	(0.003)
Log HH size × Distance to town	0.001	−0.002	0.001	0.002⁎	0.004⁎⁎
	(0.002)	(0.003)	(0.001)	(0.001)	(0.002)

Log HH size	0.610⁎⁎⁎	0.627⁎⁎⁎	0.730⁎⁎⁎	0.609⁎⁎⁎	0.363⁎⁎⁎
	(0.091)	(0.088)	(0.133)	(0.086)	(0.075)
Distance to market (km)^a^	0.003	0.006	−0.008	0.003	0.008⁎⁎⁎
	(0.003)	(0.012)	(0.012)	(0.002)	(0.003)
Log HH size × Distance to market	0	0.005	0	0	−0.002
	(0.001)	(0.008)	(0.002)	(0.001)	(0.002)

Log HH size	0.563⁎⁎⁎	0.661⁎⁎⁎	0.762⁎⁎⁎	0.562⁎⁎⁎	0.330⁎⁎⁎
	(0.072)	(0.068)	(0.073)	(0.062)	(0.058)
Distance to road (km)	−0.005	0.016⁎	−0.019⁎	−0.002	0.008
	(0.007)	(0.009)	(0.012)	(0.003)	(0.007)
Log HH size × Distance to road	0.003	0.001	−0.002	0.003⁎	−0.003
	(0.003)	(0.005)	(0.004)	(0.002)	(0.005)

Log HH size	0.685⁎⁎⁎	0.599⁎⁎⁎	0.606⁎⁎⁎	0.571⁎⁎⁎	0.296⁎⁎⁎
	(0.085)	(0.092)	(0.130)	(0.051)	(0.053)
Distance to capital (km)	0.001	0.001	−0.003	−0.001	0.003
	(0.003)	(0.004)	(0.011)	(0.001)	(0.002)
Log HH size × Distance to capital	0	0.001	0.003	0.001⁎⁎	0
	(0.000)	(0.002)	(0.002)	(0.000)	(0.001)

*Notes:* All regressions include same location fixed effects as previous tables; Standard errors clustered at level of FE; sample weights used; dependent variable in all regressions is the log of total labor demand on farm; each row group of coefficients is from a separate regression.

a Malawi the ’distance to market’ variable is measured as the distance to the nearest ADMARC facility.

⁎⁎⁎ Significant at 1%.

⁎⁎ Significant at 5%.

⁎ Significant at 10%.

**Table 9:** Log household size across agro-ecological zones.

		Warm/arid	Warm/semi-arid	Warm/sub-humid	Warm/humid	Cool/arid	Cool/semi-arid	Cool/sub-humid	Cool/humid
Ethiopia	Count	34	133	101	7	1	868	1102	511
	Log HHS (mean)	1.28	1.11	1.10	0.97	0.69	1.17	1.19	1.15
	Log HHS (sd)	0.41	0.48	0.48	0.49		0.46	0.44	0.47

Malawi	Count		1191	789			271	305	
	Log HHS (mean)		0.85	0.85			0.88	0.94	
	Log HHS (sd)		0.45	0.46			0.43	0.47	

Niger	Count	416	1767						
	Log HHS (mean)	0.94	1.05						
	Log HHS (sd)	0.42	0.47						

Tanzania	Count		168	1355	28		88	658	45
	Log HHS (mean)		0.94	1.04	0.96		1.04	1.04	1.05
	Log HHS (sd)		0.47	0.50	0.51		0.55	0.48	0.42

Uganda	Count			62	1064			321	567
	Log HHS (mean)			1.21	1.25			1.22	1.20
	Log HHS (sd)			0.52	0.57			0.55	0.59

**Table 10:** OLS results allowing for heterogeneity by agro-ecological zone.

	Ethiopia	Malawi	Niger	Tanzania	Uganda
*Dependent variable: Log labor demand*					
Log acres owned	0.192⁎⁎⁎	0.120⁎⁎⁎	0.092⁎⁎⁎	0.159⁎⁎⁎	0.143⁎⁎⁎
	(0.034)	(0.013)	(0.012)	(0.015)	(0.015)
Log median wage	−0.029	−0.143⁎⁎	−0.255⁎⁎	−0.123⁎	−0.029
	(0.065)	(0.058)	(0.107)	(0.069)	(0.047)
Log HH size	0.533⁎⁎⁎	0.544⁎⁎⁎	0.752⁎⁎⁎	0.383⁎⁎⁎	0.282⁎⁎⁎
	(0.078)	(0.083)	(0.072)	(0.073)	(0.056)
AEZ T, W, A	−0.333		0.390⁎⁎⁎		
	(0.328)		(0.139)		
AEZ T, W, A × Log HH size	−0.474⁎⁎⁎		−0.066		
	(0.140)		(0.106)		
AEZ T, W, S-A	−0.148			−0.440⁎⁎⁎	
	(0.735)			(0.166)	
AEZ T, W, S-A × Log HH size	0.152			0.229	
	(0.540)			(0.141)	
AEZ T, W, H	−0.901			−0.925⁎⁎⁎	
	(0.754)			(0.203)	
AEZ T, W, H × Log HH size	0.036			0.533⁎⁎⁎	
	(0.405)			(0.180)	
AEZ T, W, S-H		−0.135			0.185
		(0.109)			(0.201)
AEZ T, W, S-H × Log HH size		0.048			−0.029
		(0.107)			(0.147)
AEZ T, C, A	−0.083				
	(0.214)				
AEZ T, C, A × Log HH size	0.135				
	(0.137)				
AEZ T, C, S-A		−0.065		−0.382	
		(0.157)		(0.272)	
AEZ T, C, S-A × Log HH size		0.104		0.402⁎⁎	
		(0.129)		(0.175)	
AEZ T, C, H	−0.194			−1.185⁎⁎⁎	−0.085
	(0.217)			(0.316)	(0.122)
AEZ T, C, H × Log HH size	−0.103			0.695⁎⁎⁎	0.044
	(0.120)			(0.259)	(0.091)
AEZ T, C, S-H		−0.169		−0.483⁎⁎⁎	−0.133
		(0.151)		(0.128)	(0.106)
AEZ T, C, S-H × Log HH size		0.189		0.205⁎	−0.049
		(0.156)		(0.110)	(0.079)

R-squared	0.21	0.165	0.24	0.248	0.206
N	2499	2556	2183	2346	2047

St. errors clustered at EA level; sample weights used; for agro-ecological zones, T = “Tropics”, W = “Warm”, C = “Cool”, A = “Arid”, S-A = “Semiarid”, H = “Humid”, S-H = “Subhumid”; most common AEZ is omitted in each regression, which is: TCS-H for Ethiopia, TWS-A for Malawi, TWS-A for Niger, TWS-H for Tanzania, TWH for Uganda; all regressions control for share in HH population of prime age males, prime age females, and elderly females.

⁎⁎⁎ Significant at 1%.

⁎⁎ Significant at 5%.

⁎ Significant at 10%.

**Table 11:** OLS results, key coefficients from additional robustness checks.

		Ethiopia	Malawi	Niger	Tanzania	Uganda
*Dependent variable: Log labor demand*						
Above median expenditure HHs	Log HH size	0.551⁎⁎⁎	0.665⁎⁎⁎	0.790⁎⁎⁎	0.518⁎⁎⁎	0.324⁎⁎⁎
		(0.079)	(0.078)	(0.097)	(0.078)	(0.048)

Below median expenditure HHs	Log HH size	0.736⁎⁎⁎	0.700⁎⁎⁎	0.757⁎⁎⁎	0.683⁎⁎⁎	0.326⁎⁎⁎
		(0.086)	(0.073)	(0.099)	(0.069)	(0.056)

Head education	Log HH size	0.778⁎⁎⁎	0.588⁎⁎⁎	0.805⁎⁎	0.587⁎⁎⁎	0.364⁎⁎⁎
		(0.097)	(0.093)	(0.395)	(0.110)	(0.094)
	Head education (years)	0.046	−0.03		−0.011	−0.014
		(0.074)	(0.024)		(0.024)	(0.017)
	Head education (years) × Log HH size	−0.032	0.026		0.025	0.001
		(0.054)	(0.023)		(0.022)	(0.010)
	Head educ: none (=1) × Log HH size			−0.007		
				(0.595)		
	Head educ: primary (=1) × Log HH size			−0.169		
				(0.656)		
	Head educ: some sec. (=1) × Log HH size			−0.115		
				(0.638)		

Controls for soil type	Log HH size		0.619⁎⁎⁎	0.719⁎⁎⁎	0.556⁎⁎⁎	0.272⁎⁎⁎
			(0.047)	(0.076)	(0.050)	(0.045)

*Notes:* All regressions include same location fixed effects as previous tables; Standard errors clustered at level of FE; sample weights used; each row group represents a separate regression; dependent variable in all regressions is the log of total labor demand on farm; above/below median expenditure refers to expenditure per capita.

⁎⁎⁎ Significant at 1%.

⁎⁎ Significant at 5%. ^∗^ Significant at 10%.

## Footnotes

1 See Sheahan and Barrett (2014), Palacios-Lopez et al., 2015, Deininger et al., 2015 for detailed analyses of these related markets using data from the same survey project that we use.

2 There is also a structural approach to the study of the separable household model, involving estimation of production functions and comparison of the marginal product of inputs to their market prices (Jacoby, 1993, Skoufias, 1994, Barrett et al., 2008, Le, 2010). We focus on the reduced form approach because it lends itself more readily to interpretation as a specific test of market failures.

3 The natural next step is to ask whether we can use additional tests to determine which markets are the source of the underlying failure. That is the subject of ongoing work.

4 The canonical reference is Singh et al. (1986). Lau et al. (1978) and Benjamin (1992) were also helpful in developing the model outlined here.

5 Harvest labor is included in total labor demand for households in Uganda, because hired labor in the Uganda data is not disaggregated by activity.

6 See the project website for additional details: http://go.worldbank.org/OQQUQY3P70.

7 For Ethiopia, Malawi, and Tanzania we use only the primary crop listed on the plot, ignoring inter-cropping. However, the Uganda survey provides data on the percentage of land allocated to each crop at the plot level, and we use this information to distribute plot-level acreage to every crop listed on each plot.

8 The definition of a “plot” varies between surveys, so that this statistic is not directly comparable across countries.

9 For the summary statistics we define as cash crops all non-food crops, including tobacco, cotton, and sisal, as well as coffee and tea.

10 The estimator for each of these figures is a local mean smoother using an Epanechnikov kernel and a bandwidth chosen using the Stata default optimal rule-of-thumb.

11 For households in Malawi the only available distance measure for markets was distance (km) to an ADMARC center.

12 For Ethiopia in Table 10, an F-test of the null hypothesis that the sum of the coefficients on “Log HH size” and “AEZ T, W, A × Log HH size” is zero returns an F value of 0.26, with a p-value of 0.61.

## References

[b0005] Barrett C. (1996). On price risk and the inverse farm size-productivity relationship. J. Dev. Econ..

[b0010] Barrett C., Sherlund S., Adesina A. (2008). Shadow wages, allocative efficiency, and labor supply in smallholder agriculture. Agric. Econ..

[b0015] Benjamin D. (1992). Household composition, labor markets, and labor demand: testing for separation in agricultural household models. Econometrica.

[b0020] Berg E. (2013). Are poor people credit-constrained or myopic? Evidence from a South African panel. J. Dev. Econ..

[b0025] Binswanger H.P., Rosenzweig M.R. (1986). Behavioural and material determinants of production relations in agriculture. J. Develop. Stud..

[b0030] Binswanger-Mkhize, Hans P., Savastano, Sara, 2014. Agricultural intensification: the status in six African countries. World Bank Policy Research Working Paper 7116. 10.1016/j.foodpol.2016.09.021 PMC5384439 28413244

[b0035] de Janvry A., Fafchamps M., Sadoulet E. (1991). Peasant household behavior with missing markets: some paradoxes explained. Econ. J..

[b0040] Deininger, K., Xia, F., Savastano, S., 2015. Smallholders’ Land Ownership and Access in Sub-Saharan Africa. World Bank Policy Research Working Paper 7285.

[b0045] Doss C.R., Morris M. (2000). How does gender affect the adoption of agricultural innovations?. Agric. Econ..

[b0050] Fafchamps Marcel (2004). Market Institutions in Sub-Saharan Africa: Theory and Evidence.

[b0055] Feder G. (1985). The relation between farm size and farm productivity: the role of family labor, supervision and credit constraints. J. Develop. Econ..

[b0060] Holden Stein T., Binswanger Hans P. (1998). Small farmer decision-making market imperfections, and natural resource management in developing countries.

[b0065] Jacoby H. (1993). Shadow wages and peasant family labor supply: an econometric application to the Peruvian Sierra. Rev. Econ. Stud..

[b0070] Karlan D., Osei R., Osei-Akoto I., Udry C. (2014). Agricultural decisions after relaxing credit and risk constraints. Quart. J. Econ..

[b0075] LaFave D., Thomas D. (2014). Farms, Families, and Markets: New Evidence on Agricultural Labor Markets (No. w20699).

[b0080] Lau L.J., Lin W.-L., Yotopoulos P.A. (1978). The linear logarithmic expenditure system: an application to consumption-leisure choice. Econometrica.

[b0085] Le K. (2010). Separation hypothesis tests in the agricultural household model. Am. J. Agric. Econ..

[b0090] Palacios-Lopez, A., Christiaensen, L., Kilic, T., 2015. How much of the labor in African agriculture is provided by women? World Bank Policy Research Working Paper 7282. 10.1016/j.foodpol.2016.09.017 PMC5384444 28413246

[b0095] Ricker-Gilbert, J., Jayne, T., Black, J., 2009. Does Subsidizing Fertilizer Increase Yields? Evidence from Malawi. In: Presented at the 2009 AAEA Annual Meeting, Milwaukee, WI.

[b0100] Sheahan M. (2011). Analysis of Fertilizer Profitability and Use in Kenya.

[b0105] Sheahan, Megan, Barrett, Christopher B., 2014. Understanding the agricultural input landscape in sub-Saharan Africa: recent plot, household, and community-level evidence. World Bank Policy Research Working Paper 7014.

[b0110] Singh I., Squire L., Strauss J. (1986). Agricultural Household Models: Extensions, Applications, and Policy.

[b0115] Skoufias E. (1994). Using shadow wages to estimate labor supply of agricultural households. Am. J. Agric. Econ..

[b0120] Stifel D., Minten B. (2008). Isolation and agricultural productivity. Agric. Econ..

[b0125] Udry C. (1996). Gender, agricultural production, and the theory of the household. J. Political Econ..

[b0130] Udry C., Ranis G., Raut L.K. (1999). Efficiency in market structure: testing for profit maximization in African Agriculture. Trade, Growth and Development: Essays in Honor of T.N. Srinivasan.

[b0135] World Bank, 2008. World Development Report: Agriculture for Development.

